# The Risk Factors for Failure of an Upper Extremity Replantation: Is the Use of Cigarettes/Tobacco a Significant Factor?

**DOI:** 10.1371/journal.pone.0141451

**Published:** 2015-10-29

**Authors:** Ji-Yin He, Shih-Heng Chen, Tsu-Min Tsai

**Affiliations:** 1 Department of Plastic Surgery, Renji Hospital, School of Medicine, Shanghai Jiaotong University, Shanghai, China; 2 Department of Plastic and Reconstructive Surgery, Chang Gung Memorial Hospital, Chang Gung University, College of Medicine, Taipei, Taiwan; 3 Christine M. Kleinert Institute for Hand and Microsurgery, Louisville, Kentucky, United States of America; Peking Union Medical College Hospital, CHINA

## Abstract

**Background:**

The purpose of this study was to explore the potential risk factors associated with the failure of an upper extremity replantation with a focus on cigarette or tobacco use.

**Patients and Methods:**

A cohort of 102 patients with 149 replants (6 extremities, 143 digits) and a mean age of 41 years (range 5 to 72 years) was enrolled in this study. The data collected included age, gender, tobacco/cigarettes use, trauma mechanism, underlying disease (e.g., hypertension (HTN), diabetes mellitus (DM), etc.), and vein graft use. An analysis with a multivariable regression was conducted to identify the risk factors of replant failure and their respective odds ratios (ORs).

**Results:**

Multilevel generalized linear mixed models (GLMMs) with a binomial distribution and logit link showed that smoking did not increase the risk of replant failure (p = 0.234). In addition, the survival of replants was not affected by DM or HTN (p = 0.285 and 0.938, respectively). However, the replantation results were significantly affected by the age of the patients and the mechanism of injury. Patients older than 50 years and those with avulsion or crush injuries tended to have a higher risk of replant failure (OR = 2.29, 6.45, and 5.42, respectively; p = 0.047, 0.028, and 0.032, respectively).

**Conclusions:**

This study showed that the use of cigarettes/tobacco did not affect the replantation outcome. The main risks for replant failure included being older than 50 years and the trauma mechanism (avulsion or crush injuries).

## Introduction

Smoking causes many well-known deleterious effects. Smoking may impair the progression of wound healing. More specifically, smoking has been implicated in the failure of regional and free flaps [1,2]. There is ample experimental evidence indicating that tobacco is deleterious to free tissue transfer and microcirculation. Experiments with rats have shown that smoking impairs cutaneous microcirculation and increases the loss of random-pattern skin flaps [3–6]. Black [7] demonstrated that nicotine magnifies the vasoconstrictive effect of norepinephrine and impairs endothelium-dependent skin vasorelaxation in isolated perfused human skin flaps.

However, the negative effects of tobacco use do not appear to apply to all microvascular procedures. Nhabedian [8] reviewed factors associated with anastomotic failure after microvascular reconstruction of the breast in 198 women and found that tobacco use was not a risk factor for flap failure. Although cigarette/tobacco use is considered a relative contraindication of replantation by many hand surgeons [9,10], no clinical study has specifically focused on the effects of tobacco or nicotine on replantation. Thus, this retrospective review was proposed to identify the effects, if any, of cigarette/tobacco use on the outcome of replantation after traumatic amputations of an upper extremity in addition to identifying other possible risk factors of replant failure.

## Patients and Methods

After obtaining IRB approval (University of Louisville IRB), a retrospective chart review was conducted on patients who underwent an upper extremity replantation (fingers, thumbs, hands or arms) at the Christine M. Kleinert Institute for Hand and Microsurgery, Louisville, KY, USA, from 2007 to 2012. Simple revascularizations with or without vein grafts were excluded from our study.

Data from 102 patients that included 149 replantations were collected. (Informed consent was not required by the participants or the caregivers of the children because the patient records and information were anonymized and de-identified prior to any analyses). Of the patients, 91 were males, and 137 finger replantations were included. The age at the time of injury ranged between 5 and 72 years old, with a mean value of 40.71 ± 15.89 years old. In total, 67.8% of the replantations were performed on patients younger than 50, and 32.2% were performed on patients older than 50 years. During our analysis, receiver operating characteristic (ROC) curves were used to determine an appropriate point at which to differentiate the ORs of the cases versus the control subjects. In our cohort, when the cut point was set at 50 years to compare the ORs of the patients older than 50 years versus those younger than 50 years, the discriminating power was significant.

The survival of each replanted extremity was assessed at the time of suture removal (12–14 days after surgery). Failure was defined as a loss of capillary refill or any sign of partial/total necrosis. Patients who were former smokers but who had quit smoking more than half a year before replantation were considered non-smokers. The smoking status and underlying medical diseases were extracted from an anesthesia consult form.

To identify the risk factors for a replant failure, a multivariable regression was performed using the analyzed factors, including age, gender, cigarette/tobacco use, amputation mechanism (crush, saw, avulsion, guillotine), underlying diseases (hypertension (HTN), diabetes mellitus (DM), etc.), and vein graft use. An additional analysis was performed between the smokers and non-smokers to compare their demographic data and replant failure.

### Statistical analysis

We applied multilevel generalized linear mixed models (GLMMs) with a binomial distribution and logit link to assess the potential factors that were independently associated with the failure of replantation. Their respective odds ratios (ORs) and 95% confidence intervals (CIs) were calculated. Descriptive analyses were conducted for the demographic data. Tests for associations between categorical variables were based on a chi-squared test or Fisher’s exact test, depending on which test was more appropriate. Differences were considered significant if they had a p-value of 0.05 or less. All of the statistical analyses were performed using the SAS System for Windows Version 10.0 (SAS Institute Inc., Cary, NC, USA).

## Results

Of the 102 patients, 15 had DM, and 26 had HTN. Fifty-two patients were smokers or tobacco chewers. Most of the replantations (n = 143, 95.9%) were at the flexor zone II level or more distal. The remaining 6 were at the flexor zone III level. In the entire cohort, 67 replantations failed for a failure rate of 45%. In total, 56 fingers from 34 patients required vein grafts during surgery. The trauma mechanisms for these fingers were as follows: 12 were avulsion injuries, 15 were crush injuries, 18 were saw injuries, and 11 were guillotine-type injuries. The failure rates were 75% for avulsion injuries, 46.7% for crush injuries, 38.9% for saw injuries, and 27.3% for guillotine-type injuries. These data are summarized in Table 1.

**Table 1:** Basic data of the 149 replanted fingers/extremities.

Characteristics	Total	
	N	Percentage (%)
**Gender**		
Male	137	92
Female	12	8
**Age group (year)**		
< 50	101	67.8
> 50	48	32.2
**Location**		
Forearm	6	4.1
Finger	143	95.9
**Injury type**		
Crush	29	19.5
Saw	58	38.9
Avulsion	21	14.1
Guillotine	40	26.8
Gloving	1	0.7
**Diabetes mellitus**		
Yes	15	10.1
No	134	89.9
**Hypertension**		
Yes	26	17.4
No	123	82.6
**Graft**		
Yes	56	37.6
No	93	62.4

### Risk factors for failure of replantation

The multivariable regression revealed that cigarette smoking/tobacco chewing did not affect the outcome of replantation (p = 0.234). Neither DM (p = 0.285) nor HTN (p = 0.938) affected the results of replantation. The significant risk factors for replant failure included older age (> 50 years), avulsion injuries, and crush injuries. Being older than 50 years had an OR of 2.29 with a 95% CI of 1.52–7.62 (p = 0.047). The patients with avulsion and crush injuries (OR = 6.45, 95% CI = 1.23–33.91, p = 0.028; and OR = 5.42, 95% CI = 1.15–15.42, p = 0.032, respectively), but not saw injuries (OR = 1.18, 95% CI = 0.21–6.69), were more likely to fail in replantation. The use of vein grafts during a replantation does not increase the risk of failure (p = 0.312). The results of the statistical analysis are summarized in Table 2.

**Table 2:** Multivariate analysis of the potential influential factors for the failure of replantation.

Factors	N* (%**)	OR (95% CI)	P-value
**Gender**			
Male	59 (43.1)	1.00	
Female	8 (66.7)	2.01 (0.33–12.69)	0.453
**Age group (year)**			
< 50	40 (39.6)	1.00	
> 50	27 (56.2)	2.29 (1.52–7.62)	0.047
**Cigarette/tobacco**			
Yes	29 (39.7)	1.00	
No	38 (50.0)	1.85 (0.67–5.14)	0.234
**Injury type** ***			
Guillotine	11 (27.5)	1.00	
Saw	20 (34.5)	1.18 (0.21–6.69)	0.854
Crush	19 (65.6)	5.42 (1.15–15.42)	0.032
Avulsion	16 (76.2)	6.45 (1.23–33.91)	0.028
**Diabetes mellitus**			
Yes	5 (33.3)	1.00	
No	62 (46.3)	2.42 (0.48–12.36)	0.285
**HTN**			
Yes	12 (46.2)	1.00	
No	55 (44.7)	0.94 (0.25–3.75)	0.938
**Graft**			
Yes	25 (44.6)	1.00	
No	42 (45.2)	1.389 (0.59–5.10	0.312

OR: odds ratio. CI: confidence interval.

*N: number of failed replantations.

**%: percentage of failed replantations (failed/ failed+survived).

***One case with a degloving injury was considered an avulsion injury during the statistical analysis.

### Comparison between the smoking and non-smoking groups

In total, 52 patients were smokers or tobacco chewers. In the smoking group, 4 patients had DM and 11 patients had HTN. In the non-smoking group, 11 patients had DM and 15 patients had HTN. There was no statistical difference between the DM (p = 0.068) or HTN (p = 0.453) incidences in the smoking and non-smoking groups.

The percentages of the different mechanisms of injury in the smoking group were 19.2%, 49.3%, 6.8%, and 24.7% for crush, saw, avulsion, and guillotine injuries, respectively, compared to 19.7%, 28.9%, 21.1%, and 28.9% in the non-smoking group, respectively. The percentage of saw injuries in the smoking group was higher than in the non-smoking group whereas the percentage of avulsion injuries in the smoking group was lower than in the non-smoking group (Table 3). There was no significant difference between the smokers and non-smokers in regard to vein-graft use during replantation (p = 0.849). There was also no significant difference (p = 0.25) in the failure rate between the smoking (failure rate = 39.7%) and non-smoking groups (failure rate = 50%). In total, 4 patients had a postoperative infection. Three of these patients were smokers, and 1 was a non-smoker. All of the patients eventually healed after a dressing change. The chi-square comparison between the smokers and non-smokers is summarized in Table 3.

**Table 3:** Comparison of the sociodemographic characteristics between smokers and non-smokers*.

Characteristics	Smoker		Non-smoker		p-value
	N	Percentage	N	Percentage	
**Total**	73		76		
**Gender**					
Male	70	95.9%	67	88.2%	0.083
Female	3	4.1%	9	11.8%	
**Age (years)**					
< 50	47	64.4%	54	71.1%	0.384
> 50	26	35.6%	22	28.9%	
**Location**					
Forearm	3	4.1%	3	3.9%	0.960
Finger	70	95.9%	73	96.1%	
**Injury type**					0.033
Crush	14	19.2%	15	19.7%	1.00
Saw	36	49.3%	22	28.9%	0.0122
Avulsion	5	6.8%	16	21.1%	0.0175
Guillotine	18	24.7%	22	28.9%	0.5837
Degloving	0	0	1	1.3%	1.00
**Diabetes mellitus**					
Yes	4	5.5%	11	14.5%	0.068
No	69	94.5%	65	85.5%	
**HTN**					
Yes	11	15.1%	15	19.7%	0.453
No	62	84.9%	61	80.3%	
**Graft**					
Yes	28	38.4%	28	36.8%	0.849
No	45	61.6%	48	63.2%	
**Replant survival**					
Yes	44	60.3%	38	50%	0.250
No	29	39.7%	38	50%	
**PostOP Infection**					
Yes	3	4.1%	1	1.3%	0.292
No	70	95.9%	75	98.7%	

*Chi-square data

## Discussion

Smokers have severely impaired peripheral microcirculation. The negative effects of smoking on the microvasculature are attributed to the disruption of the balance between complement activation and regulation at the endothelial cell surface. This may affect vascular injury. Other studies have investigated whether smoke can cause endothelial dysfunction [11–13]. However, all of these studies were performed on normal extremities. It is not known how replanted tissues, which have suffered from a period of ischemia and potentially suboptimal blood flow and re-innervation after a replantation, are affected by cigarette/tobacco use. Based on the aforementioned studies, it is reasonable to believe that smoking would be harmful to the survival of a replanted digit or extremity. However, some scientific evidence suggests that tobacco use does not always result in significant vascular impairment. Studies on reconstructive microsurgeries have shown that there is no significant drop in dermal perfusion after chronic tobacco consumption [13,14].

Our results showed that the use of cigarettes/tobacco did not increase the failure rate of replantation. Thus, it might be inappropriate to consider smoking a contraindication for replantation. However, four cases were complicated with postoperative infection. Three of these patients did not quit smoking postoperatively. Although we did not compare “smokers who continued smoking after replant” versus “smokers who ceased smoking after replant” in assessing the replant failure and complication rates in this study, other studies have shown that smoking may increase the risk of infection [15]. Therefore, we still suggest that our patients quit smoking for at least 3 months after replantation surgery.

Other studies [16,17] have also previously mentioned that age might be an important factor that affects the survival of replantation. These studies purposely divided the patients into certain age groups. The statistical results indicated that age had no significant effect on replantation. Our analysis showed that being older than 50 years of age has an increased risk of replant failure. The patients older than 50 years had a replant failure OR of 1.96. Although this finding might not affect the clinical decision of performing a replantation, it offers evidence to inform the patient and family that there is an increased risk of replant failure in patients older than 50 years.

Another factor that significantly affected the outcome of replantation was the mechanism of injury. The multivariable analysis showed that the chance of failure was higher in the crush and avulsion cases compared to the saw and guillotine injuries, which was similar to the study by Fufa [18]. The failure rate was 84% in crush injuries and 85% in avulsion injuries. These injuries are more complicated because the trauma zone might be underestimated. The arteries and veins of the entire amputee, including the microvasculature, might be severely contused with such a mechanism. Particularly in the avulsion cases, the vessels are avulsed from the proximal stump, resulting in a complexity of replantation. In our practice, the bone was generally shortened approximately 0.5 to 1 cm to obtain relatively healthy tissues, particularly the arteries and veins. If vascular anastomosis could not be performed without tension, a vein graft would be used, as needed. However, it is not uncommon to have multiple anastomosis attempts with longer vein grafts because of the difficulty in clinically defining the actual trauma zone. Heparin was generally systemically applied if vein grafts were used. In this series, the use of vein graft did not statistically affect the success rate of the replant.

The success rate of replantation in our series was 55%, which was similar to the results of a recent publication by other two level-I trauma centers (57%) [18]. The lower success rate in our study may be because our series comprised a higher percentage of crush and avulsion injuries. Our statistical data will also help physicians inform patients and their families that the success of replantation is significantly affected by the trauma mechanism.

This study has several limitations. First, the status of the surgeon could not be evaluated. Although all of our surgeons had been well trained in microsurgery, there are many factors that affect a surgeon’s performance, such as overworking. Second, we calculated the failure rate of replantation based on the number of replanted digits or extremities rather than on the patient number. However, multiple-digit replantation could be a potential risk factor for failure compared to a single-digit replantation, which was not shown in this series.

## Conclusions

In this study, being older than 50 years of age, avulsion injuries, and crush injuries had a negative effect on the success of replantation surgeries. However, cigarette smoking and tobacco chewing did not affect the outcome of replantation.
